# Integrated Assessment of the Cardiotoxic and Neurobehavioral Effects of 3,4-Methylenedioxypyrovalerone (MDPV) in Zebrafish Embryos

**DOI:** 10.3390/ijms27010059

**Published:** 2025-12-20

**Authors:** Ouwais Aljabasini, Niki Tagkalidou, Juliette Bedrossiantz, Eva Prats, Raul Lopez-Arnau, Demetrio Raldua

**Affiliations:** 1 Institute for Environmental Assessment and Water Research (IDAEA-CSIC), Jordi Girona, 18, 08034 Barcelona, Spain; ouwais.aljabasini@idaea.csic.es (O.A.); niki.tagkalidou@idaea.csic.es (N.T.); jbdqam@cid.csic.es (J.B.); 2 Research and Development Center (CID-CSIC), Jordi Girona, 18, 08034 Barcelona, Spain; epmbmc@cid.csic.es; 3 Department of Pharmacology, Toxicology and Therapeutic Chemistry, Faculty of Pharmacy and Food Sciences, Pharmacology Section, Institute of Biomedicine (IBUB), University of Barcelona, 08028 Barcelona, Spain; raullopezarnau@ub.edu

**Keywords:** MDPV, synthetic cathinones, zebrafish embryos, cardiotoxicity, prepulse inhibition, locomotor activity, dopaminergic signaling, gene expression

## Abstract

Synthetic cathinones such as 3,4-methylenedioxypyrovalerone (MDPV) are potent psychostimulants with high abuse potential, yet their systemic toxicity and neurobehavioral effects remain poorly characterized during early development. Using *Danio rerio* (zebrafish) embryos and larvae, we performed an integrated assessment of the cardiotoxic, behavioral, and molecular effects of MDPV. Acute exposure of 3 days post-fertilization (dpf) embryos produced a marked, concentration-dependent bradycardia and atrioventricular (AV) conduction block, leading to reduced ventricular activity and complete AV dissociation at the highest concentrations (EC_50_ = 228 µM). Quantitative analysis of ventricular motion revealed a significant decrease in cardiac output (CO) at all tested concentrations and a reduction in ejection fraction (EF) only at 480 µM, while fractional shortening (FS) and stroke volume (SV) remained unchanged, indicating predominant chronotropic and conduction effects with secondary contractile impairment. In 5 dpf larvae, MDPV caused a sustained, concentration-dependent decrease in basal locomotor activity (EC_50_ = 2.51 µM) but did not affect prepulse inhibition (PPI) of the acoustic startle response (ASR), unlike dextroamphetamine, which enhanced PPI via dopaminergic D_2_ receptor activation. Short-term (2 h) exposure of 3 dpf embryos to 0.4–400 µM MDPV induced transcriptional changes in dopaminergic and stress-responsive genes, whereas expression of major repolarizing potassium channel genes (*kcnh6a* and *kcnq1*) remained unaltered. Collectively, these results demonstrate that MDPV exerts potent negative chronotropic effects likely through direct functional interference with cardiac repolarization, while neurobehavioral effects occur at concentrations nearly two orders of magnitude lower than cardiotoxic thresholds, supporting zebrafish as a predictive model for the integrative assessment of psychostimulant toxicity.

## 1. Introduction

3,4-Methylenedioxypyrovalerone (MDPV) is a new psychoactive substance (NPS), belonging to the class of synthetic cathinones, which is structurally related to pyrovalerone and 3,4-methylenedioxymethamphetamine (MDMA) [1,2]. MDPV acts primarily as a potent dopamine (DA) and norepinephrine (NE) transporter (DAT and NET, respectively) blocker, with negligible activity at the serotonin transporter (SERT) [1,3]. By elevating synaptic catecholamines, particularly DA in reward-related regions, MDPV produces strong psychostimulant and reinforcing effects [4]. Moreover, DAT/NET blockade contributes to adverse physiological consequences, including severe agitation, hypertension, hyperthermia, seizures, and cardiac arrhythmias [4,5,6,7]. Since its emergence as a recreational drug, MDPV has been implicated in numerous cases of acute intoxication and death [5,8,9]. Despite these alarming reports, the precise mechanisms by which MDPV induces cardiotoxic effects are still not fully understood.

The cardiovascular system is particularly vulnerable to psychostimulants that enhance catecholaminergic activity. Increased sympathetic drive and catecholamine release can result in vasoconstriction, hypertension, tachycardia, arrhythmias, and, in severe cases, cardiac arrest [1,10]. In humans and mammalian models, these compounds often induce pronounced hyperthermia, which exacerbates metabolic stress and predisposes to rhythm disturbances [11]. Hyperthermia therefore represents a major confounding factor in assessing direct cardiotoxicity. However, specific data regarding the direct impact of MDPV on cardiac function remain scarce [7,12], underscoring the need for more comprehensive studies.

Zebrafish (*Danio rerio*) have become a highly relevant model organism for investigating the molecular and physiological effects of neuroactive and cardiotoxic compounds. Their rapid development, transparency, and strong physiological homology with mammals facilitate real-time assessment of cardiac and behavioral phenotypes [13,14]. The zebrafish heart shares remarkable structural and electrophysiological similarities with the human heart, including nodal tissue and comparable ion-channel expression [15]. Furthermore, early transparency enables non-invasive visualization of heart rate, rhythm, and conduction, facilitating the detection of arrhythmias [16,17,18]. Moreover, unlike homeothermic mammalian species, zebrafish do not develop drug-induced hyperthermia, allowing direct evaluation of intrinsic cardiotoxicity without thermal confounders [19]. Finally, at 5 days post-fertilization (dpf), zebrafish embryos exhibit robust locomotor patterns that are modulated by neuroactive substances, making them an excellent system for evaluating psychotropic or neurotoxic effects.

In this context, zebrafish provide a powerful integrative model to assess cardiac, neurobehavioral, and molecular endpoints of MDPV toxicity within a unified framework. Previous research on synthetic cathinones in zebrafish has primarily focused on behavioral alterations [20,21] or general developmental toxicity [22], whereas integrated analyses across these domains remain limited. Such integrative approaches are essential to understand coordinated neural–cardiac effects and detect early molecular signatures of toxicity.

The concentration range selected in this study (5 nM–400 µM) was based on previous work investigating the behavioral and cardiotoxic effects of synthetic cathinones in zebrafish and mammalian models [2,17]. Low micromolar concentrations correspond to levels that elicit psychostimulant-like effects via DAT blockade [2], whereas high micromolar concentrations produce conduction disturbances and arrhythmias in zebrafish embryos [17]. This range, therefore, enables simultaneous evaluation of neurobehavioral and cardiac endpoints in the same study.

Based on these considerations, we hypothesized that MDPV would (1) disrupt cardiac electrophysiology at high micromolar concentrations and (2) induce neurobehavioral alterations at substantially lower concentrations, resulting in a functional separation between neural and cardiac toxicity thresholds.

The present study aimed to provide an integrated assessment of MDPV toxicity across physiological, behavioral, and molecular domains. First, acute cardiotoxicity and lethality were evaluated in 3 dpf embryos by measuring heart rate, atrioventricular conduction, and ventricular performance (FS, SV, EF, CO)). Neurobehavioral effects were assessed by quantifying basal locomotor activity at 5 dpf and sensorimotor gating through prepulse inhibition (PPI) of the acoustic startle response (ASR) at 7 dpf, a developmental stage at which dopaminergic modulation of startle behavior is functionally established [23]. Finally, early-response gene transcription was analyzed following short-term MDPV exposure to identify molecular pathways underlying its effects.

Together, these complementary approaches define the cardiotoxic, neurobehavioral, and molecular effects of MDPV in zebrafish and identify molecular pathways associated with its toxic action. Comparison of concentrations producing locomotor suppression and atrioventricular block further defines a functional safety margin between neurobehavioral and cardiotoxic effects.

## 2. Results

### 2.1. Lethality Test

After 24 h of exposure, MDPV induced a clear concentration-dependent increase in lethality in 3 dpf zebrafish embryos (Supplementary Figure S1). At 540 and 600 µM, mortality reached 100%, while 420 and 480 µM resulted in lethality over 85% (individual data are available at Supplementary Dataset S1).

Dose–response fitting using a four-parameter logistic (4PL) model confirmed the steep concentration dependence of MDPV-induced mortality. The model yielded an LC_50_ of 343.8 µM (95% bootstrap CI: 318.1–369.4 µM) and a Hill slope of 9.81 (95% CI: 6.75–10.00), indicating a sharp transition between sublethal and lethal concentrations.

These results demonstrate that MDPV exerts a potent, sharply concentration-dependent lethality in zebrafish embryos following 24 h of exposure, with complete mortality observed at 540 µM.

### 2.2. Cardiotoxicity Assessment

#### 2.2.1. Chronotropic and Conduction Effects of MDPV

**Figure 1:**
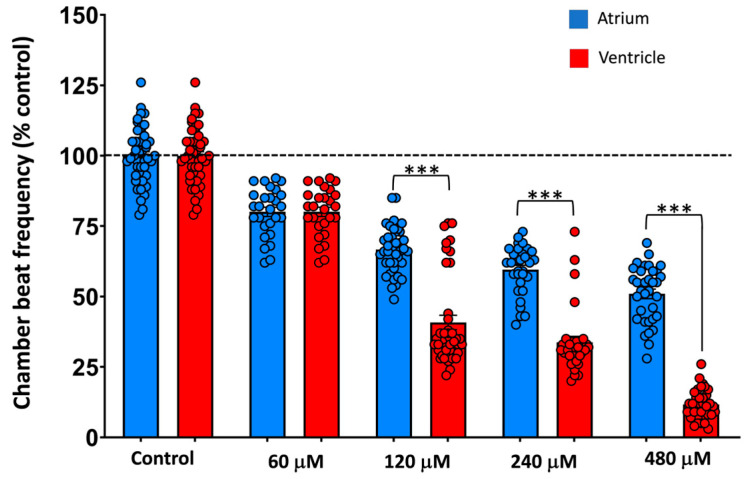
Effect of a 2 h waterborne exposure to 60–480 µM MDPV on atrial and ventricular beat frequency in 3 dpf zebrafish embryos. Data are mean ± SEM, showing all data (*N*_control_ = 47; *N*_MDPV_ (60 µM) = 27; *N*_MDPV_ (120 µM) = 40; *N*_MDPV_ (240 µM) = 31; *N*_MDPV_ (480 µM) = 34). A horizontal dashed line indicates the 100% control reference level. Differences between atrial and ventricular beat frequencies at each concentration were assessed by Student’s *t*-test. *** *p* < 0.001.

Figure 1 shows the main effects of 2 h waterborne exposure to 60–480 µM MDPV on the beat frequency of the atrium and ventricle of 3 dpf embryos (individual data are available at Supplementary Dataset S2). A mixed repeated-measures ANOVA revealed significant effects of heart chamber (*F*(1,174) = 642.42, *p* = 2.65 × 10^−60^, *ηp*^2^ = 0.79), MDPV concentration [*F*(4,174) = 355.21, *p* = 1.54 × 10^−82^, *ηp*^2^ = 0.89], and their interaction [*F*(4,174) = 122.91, *p* = 1.32 × 10^−49^, *ηp*^2^ = 0.74] on normalized beat frequencies.

**Figure 2:**
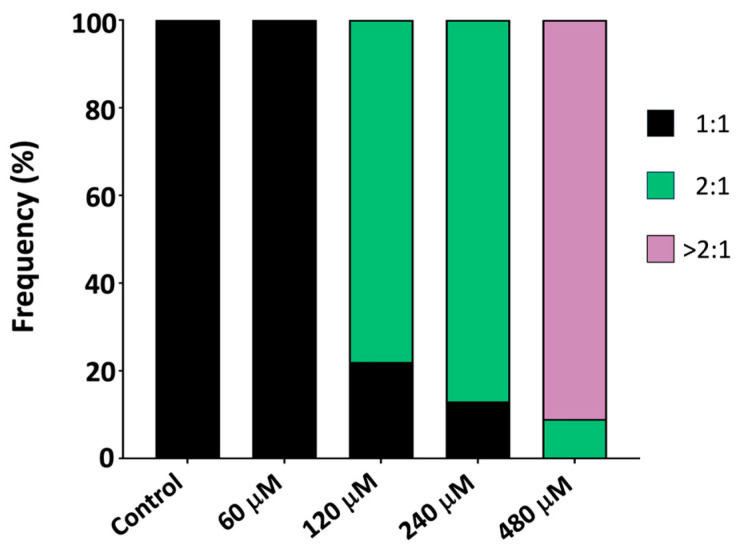
Effect of a 2 h waterborne exposure to 60–480 µM MDPV on the atrioventricular (AV) ratio (*N*_control_ = 47; *N*_MDPV_ (60 µM) = 27; *N*_MDPV_ (120 µM) = 40; *N*_MDPV_ (240 µM) = 31; *N*_MDPV_ (480 µM) = 34).

Control embryos showed the expected 1:1 atrioventricular (AV) conduction pattern (Figure 2 MDPV induced a concentration-dependent disruption of this synchrony. At 60 µM, cardiac rhythm slowed without loss of coordination, maintaining a 1:1 atrioventricular (AV) conduction ratio. From 120 µM onward, ventricular contractions became progressively dissociated from the atrial rhythm, giving rise to 2:1 AV block that advanced to higher-degree block at 240–480 µM.

Post hoc comparisons confirmed that all concentrations significantly reduced atrial beat frequency relative to controls (all *p* < 0.001), whereas ventricular rate exhibited a much steeper decline, with 480 µM producing near-complete suppression of ventricular activity (92% reduction vs. control, *p* < 0.001). These differential responses explain the marked interaction effect and demonstrate preferential vulnerability of ventricular conduction to MDPV.

To quantify the onset of conduction failure, the atrioventricular conduction ratio was converted into % AV block using % block = 100 × (1 − 1/R), where R = AV ratio. A logistic concentration–response model (Supplementary Figure S2) revealed a monotonic increase in AV block with an EC_50_ of 228.3 µM (95% bootstrap CI: 208.1–253.3 µM; Hill slope 1.45). This parameter defines the midpoint of the transition from rhythmic entrainment to high-degree AV block, confirming that MDPV induces a sharp, concentration-dependent collapse of ventricular conduction at high micromolar levels.

Together, these findings demonstrate that MDPV causes dose-dependent bradycardia driven by preferential suppression of ventricular excitability, culminating in complete AV block at the highest concentrations tested.

#### 2.2.2. Mechanical Cardiac Performance

**Figure 3:**
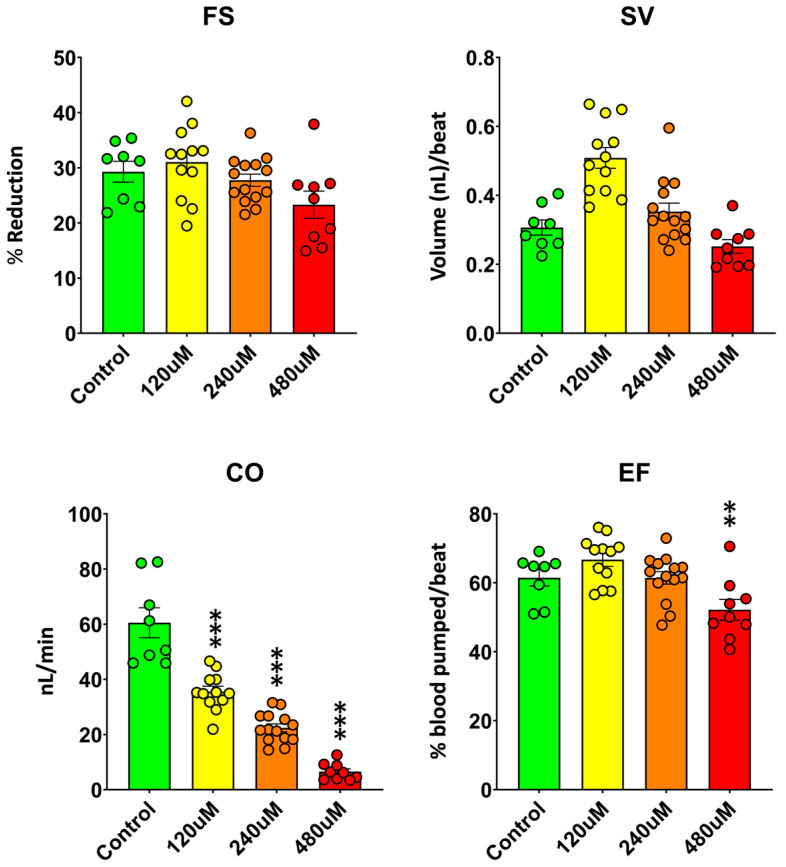
Effects of MDPV on ventricular performance in 3 dpf zebrafish embryos. Fractional shortening (FS), stroke volume (SV), cardiac output (CO), and ejection fraction (EF) were measured after 2 h exposure to 120–480 µM MDPV. Data are mean ± SEM, showing all data. One-way ANOVA with Dunnett’s post hoc test; ** *p* < 0.01 and *** *p* < 0.001 vs. Control group.

To further characterize the cardiac effects of MDPV, quantitative analysis of ventricular motion was performed to determine fractional shortening (FS), stroke volume (SV), ejection fraction (EF), and cardiac output (CO) in 3 dpf zebrafish embryos after a 2 h exposure to 120–480 µM MDPV (Figure 3). These parameters represent integrated measures of ventricular contractility and overall pump performance (individual data are available at Supplementary Dataset S3).

MDPV exposure produced a concentration-dependent decline in overall cardiac function. A one-way ANOVA revealed significant effects of treatment on CO (*F*(3, 39) = 67.6, *p* = 1.6 × 10^−15^) and EF (*F*(3, 39) = 6.9, *p* = 7.8 × 10^−4^), whereas effects on SV and FS were less pronounced. Post hoc Dunnett comparisons against controls confirmed that CO was significantly reduced at all tested MDPV concentrations (*p* < 0.001 for all), while EF was only significantly decreased at 480 µM (*p* < 0.01). FS and SV values did not differ significantly from controls (all *p* > 0.05).

The observed reduction in CO closely paralleled the concentration-dependent bradycardia and progressive atrioventricular conduction block described above, indicating that MDPV primarily affects cardiac performance through chronotropic and conduction mechanisms. The significant decrease in EF at 480 µM suggests an additional contractile impairment emerging at higher concentrations. These results demonstrate that MDPV exerts concentration-dependent negative chronotropic and, at high concentrations, inotropic effects in zebrafish embryos, leading to substantial reductions in overall cardiac output.

### 2.3. Behavioral Assessment

#### 2.3.1. Basal Locomotor Activity

Basal locomotor activity (BLA) was assessed in 5 dpf zebrafish embryos exposed to a range of concentrations from 5 nM to 5 mM MDPV. Locomotor behavior was recorded during three 15 min observation windows: 0–15 min, 45–60 min, and 105–120 min after the onset of exposure. Individual data are available at Supplementary Dataset S4.

**Figure 4:**
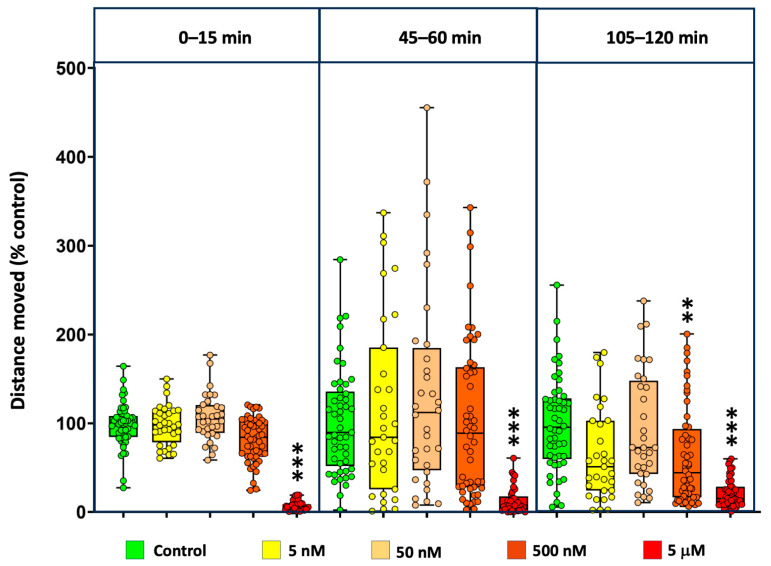
Basal locomotor activity (BLA) decreased in a time- and concentration-dependent manner in 5 dpf zebrafish embryos exposed to MDPV. Data are presented as boxplots where the box indicates the 25th and 75th percentiles, the thin line within the box marks the median, and the whiskers the maximum and minimum values, showing all data. ** *p* < 0.01, *** *p* < 0.001; Kruskal–Wallis test with Bonferroni correction.

As shown in Figure 4, MDPV exposure induced a concentration-dependent decrease in BLA relative to controls. The effect was particularly evident at the highest concentration tested (5 µM), where activity was significantly reduced in all three periods analyzed. Kruskal–Wallis analyses confirmed significant treatment effects for each observation window: 0–15 min [*H*(4) = 127.4, *p* < 0.001], 45–60 min [*H*(4) = 50.18, *p* < 0.001], and 105–120 min [*H*(4) = 54.90, *p* < 0.001]. Post hoc comparisons indicated that at the last observation window (105–120 min), only the 0.5 µM and 5 µM groups differed significantly from the control (*p* < 0.05), whereas lower concentrations (0.005 and 0.05 µM) had no detectable effect.

To further characterize the concentration–response relationship, replicate-level data from the 105–120 min interval were fitted to a four-parameter logistic (4PL) model. As shown in Supplementary Figure S3, the best-fit curve described a monotonic, sigmoidal inhibition of locomotor activity, with an estimated EC_50_ of 2.51 µM (95% bootstrap CI: 0.38–5.08 µM) and a Hill slope of −0.98. The top and bottom plateaus of the curve corresponded to approximately 80.7% and 7.3% of control activity, respectively, while the calculated EC_10_ and EC_90_ were 0.27 µM and 23.4 µM. These quantitative results are consistent with the nonparametric analyses, confirming that MDPV produces a concentration-dependent and sustained suppression of locomotor behavior, with half-maximal inhibition occurring at low micromolar concentrations.

#### 2.3.2. Prepulse Inhibition

The disruptive effects of amphetamine-type stimulants on prepulse inhibition (PPI) are well established in mammals and humans, where they are mediated by dopaminergic activation of D_2_ receptors. However, the effects of such stimulants on PPI had not been assessed in zebrafish, so dextroamphetamine was first used as a reference compound to validate the assay before assessing the potential effects of MDPV.

As shown in Supplementary Figure S4, exposure of 7 dpf zebrafish larvae to 5 µM dextroamphetamine for 30 min produced a robust increase in PPI relative to controls at both 50 ms and 500 ms prepulse–pulse intervals. To confirm the dopaminergic basis of this effect, co-exposure with the D_2_ receptor antagonist haloperidol (0.5 µM) was performed, which completely prevented dextroamphetamine-induced PPI enhancement, restoring responses to control levels.

In the 50 ms interval, the median (IQR) PPI values were 80.0% (71.4–100.0) in controls, 100.0% (100.0–100.0) in dextroamphetamine-treated larvae, and 77.8% (55.6–87.5) in the haloperidol co-treated group. In the 500 ms interval, the corresponding values were 77.1% (61.5–81.5), 100.0% (86.1–100.0), and 50.0% (33.3–77.8), respectively. Statistical analysis confirmed significant differences among the experimental groups [50 ms interval: *H*(2) = 27.93, *p* = 8.4 × 10^−7^; 500 ms interval: *H*(2) = 34.06, *p* = 4.0 × 10^−8^]. These results demonstrate that dextroamphetamine exposure enhances PPI in zebrafish larvae and that this effect is mediated by D_2_ receptors.

In contrast, MDPV exposure did not significantly alter PPI. In the 50 ms interval, median (IQR) PPI values were 80.0% (63.9–100.0) in controls, 79.2% (61.3–100.0) in 5 nM MDPV-treated larvae, 82.5% (52.6–95.7) in 500 nM MDPV-treated larvae, and 83.3% (66.7–100.0) in 5 µM MDPV-treated larvae. In the 500 ms interval, the corresponding values were 66.7% (46.4–83.9), 58.3% (36.0–71.7), 73.8% (48.3–82.8), and 82.5% (64.3–100.0), respectively (individual data are available at Supplementary Dataset S5). Statistical analysis confirmed no significant differences between control and MDPV-treated groups].

### 2.4. Gene Expression Analysis

To explore the molecular mechanisms underlying the behavioral effects of MDPV, we assessed the transcriptional response of a panel of early-response genes known to be modulated by psychostimulants such as dextroamphetamine or synthetic cathinones. A 2 h exposure period was selected to capture rapid gene expression changes induced by acute MDPV exposure. The analyzed genes included *th* (tyrosine hydroxylase), *slc6a3* (DAT), *kcnh2a* (potassium voltage-gated channel subfamily H member 2a), *kcnh6a* (hERG-like IKr), *kcnq1* (potassium voltage-gated channel subfamily Q member 1, IKs), *fosab* (v-fos murine osteosarcoma viral oncogene homolog Ab), *egr1* (early growth response 1), *npas4a* (neuronal PAS domain protein 4a), *nr4a1* (nuclear receptor subfamily 4 group A member 1), and *fkbp5* (FK506-binding protein 5).

**Figure 5:**
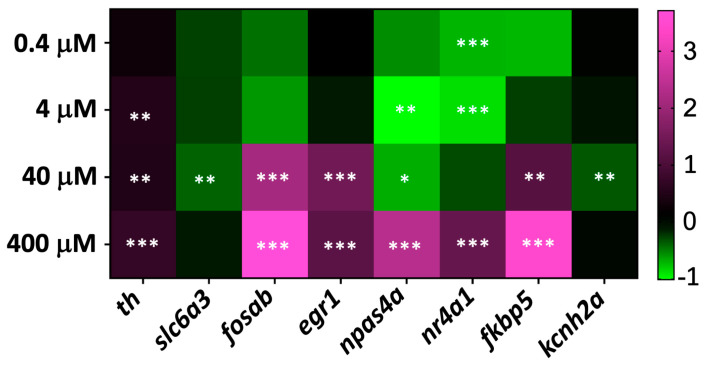
Heat map of relative gene expression (logΔΔCT) in embryos exposed to MDPV. Expression levels of selected genes after exposure to 0.4–400 µM MDPV. Colors indicate modulation relative to control (purple, up-regulation; green, down-regulation). Asterisks indicate statistically significant differences from control according to one-way ANOVA followed by Dunnett’s multiple comparison test (* *p* < 0.05, ** *p* < 0.01, *** *p* < 0.001).

Three-day-old zebrafish embryos were exposed to MDPV concentrations ranging from 0.4 to 400 µM for 2 h. As shown in Figure 5, the expression profile revealed distinct transcriptional modulation across concentrations]. Exposure to 4–400 µM MDPV led to a mild but significant upregulation of *th*, suggesting enhanced dopaminergic activity. In contrast, *slc6a3* and *kcnh2a* were significantly downregulated only at 40 µM.

Among immediate-early genes, *fosab*, *npas4a*, and *fkbp5* were markedly upregulated in the 40–400 µM range, consistent with neuronal activation and stress-related signaling. *nr4a1* displayed a biphasic response, being downregulated at low concentrations (0.4–4 µM) and upregulated at 400 µM. Similarly, *npas4a* was downregulated at 4–40 µM and strong upregulation at 400 µM.

No significant transcriptional modulation was detected for *kcnh6a* (IKr) and *kcnq1* (IKs) at any MDPV concentrations tested.

Detailed statistical results are presented in Supplementary Table S1, and the overall gene expression profiles are summarized in Figure 5.

## 3. Discussion

This study provides an integrated evaluation of the cardiotoxic and neurobehavioral effects of the cathinone 3,4-methylenedioxypyrovalerone (MDPV) in zebrafish embryos and larvae. By combining physiological, behavioral, and molecular analyses across developmental stages, we demonstrated that MDPV exerts a potent negative chronotropic effect and concentration-dependent transcriptional modulation of early-response genes, while producing no significant alterations in sensorimotor gating. These findings highlight the value of the zebrafish model for characterizing the direct and system-level consequences of emerging psychostimulants.

### 3.1. Cardiotoxicity of MDPV in Zebrafish Embryos

#### 3.1.1. Chronotropic and Conduction Effects of MDPV in Zebrafish Embryos

Psychostimulant drugs, including methamphetamine, cocaine, and synthetic cathinones, are known to induce tachycardia, conduction and repolarization disturbances, and QT interval prolongation [24,25,26,27]. Compounds that cause repolarization abnormalities in humans or mammalian models typically produce bradycardia and atrioventricular (AV) block in zebrafish embryos [17,28,29]. Consistent with this, the present study found that acute exposure to MDPV produced a marked, concentration-dependent depression of cardiac activity in 3 dpf embryos. Both atrial and ventricular beating frequencies decreased significantly, but ventricular suppression was more pronounced, resulting in AV conduction blocks at higher concentrations. These findings align with the arrhythmias and cardiac arrest reported in mammalian models [7,12], clinical intoxications [5,8], and previous zebrafish studies with MDPV [17].

The preferential vulnerability of ventricular tissue suggests interference with repolarizing potassium currents mediated by the zebrafish orthologs of KCNH2 (hERG) [28,29,30,31] or KCNQ1 channels [27], which represent known molecular targets of synthetic cathinones. Although our study did not measure ion-channel currents or protein levels, the absence of transcriptional changes in *kcnh6a* and *kcnq1* at all tested concentrations indicates that the observed conduction impairment most likely reflects direct functional interference with repolarizing potassium currents (IKr/IKs-like) rather than genomic remodeling. This interpretation aligns with previous reports showing that QT-prolonging compounds can induce bradyarrhythmia and AV block in zebrafish embryos through acute electrophysiological mechanisms without altering ion-channel expression [28,29].

The absence of hyperthermic confounding in zebrafish reinforces the interpretation that MDPV can directly impair cardiac excitability via ion-channel modulation rather than secondary thermal effects. Interestingly, a recent study using human iPSC-derived cardiomyocytes—another model that avoids hyperthermia as a confounding variable—reported that MDPV caused a concentration-dependent reduction in spike amplitude and beat rate, together with prolongation of the field potential duration (FPD), a parameter analogous to the QT interval [32]. These findings are highly consistent with those obtained in the present zebrafish model.

The 24 h LC_50_ value determined in the present study (344 µM) was higher than that previously reported by Teixidó et al. [17] (135 µM). This discrepancy may reflect differences in the developmental stages at which LC_50_ was assessed, namely 3 dpf in the current study versus 4 dpf in Teixidó et al. Although a direct experimental comparison would be informative, the present work focused specifically on 3 dpf embryos because this time point offers optimal optical transparency for cardiac imaging and precedes the establishment of significant autonomic modulation. This stage allows the integrated assessment of cardiac, behavioral, and transcriptional endpoints within a unified developmental framework. Future studies comparing LC_50_ values at 3 and 4 dpf may help resolve whether developmental maturation accounts for the observed discrepancy.

Importantly, the observed lethality at high MDPV concentrations cannot be directly ascribed to cardiac failure. At 3 dpf, zebrafish embryos rely predominantly on cutaneous gas exchange and can survive without functional circulation until approximately 7 dpf. [33]. Consequently, even though MDPV induces marked bradycardia and atrioventricular block, mortality at concentrations ≥400 µM is unlikely to result from hemodynamic collapse. Instead, lethality most likely reflects systemic cellular toxicity or metabolic disruption induced at these high exposure levels.

#### 3.1.2. Effects of MDPV on Cardiac Mechanical Performance

In addition to the pronounced bradycardia and atrioventricular conduction blocks, quantitative analysis of ventricular mechanics revealed a clear concentration-dependent reduction in cardiac output (CO), with significant decreases at all MDPV concentrations tested. Ejection fraction (EF) was also reduced at the highest concentration (480 µM), whereas fractional shortening (FS) and stroke volume (SV) remained largely unchanged relative to controls. These results indicate that MDPV primarily impairs cardiac performance through chronotropic and conduction mechanisms, while ventricular contractility is affected only at higher exposure levels.

The selective reduction in CO across all concentrations is consistent with the severe bradycardia observed, since CO integrates both stroke volume and heart rate. Even moderate slowing of cardiac rhythm or partial atrioventricular block can substantially diminish effective blood flow without requiring a parallel reduction in contractile strength. The further decline in EF at 480 µM suggests that, beyond a certain threshold, MDPV also compromises ventricular contractility, possibly through interference with ion-channel activity, particularly KCNQ1-mediated repolarizing currents, which are highly sensitive to synthetic cathinones and structurally related stimulants [27]. Notably, a significant decrease in EF is considered an indicator of heart failure [34]. Such dual chronotropic and inotropic impairment aligns with reports of arrhythmias and pump failure in mammalian models and human MDPV intoxications [7,24,25,26,27].

The apparent discrepancy between the bradycardia observed in zebrafish embryos and the tachycardia typically reported in mammals following exposure to psychostimulants such as MDPV [4,7,24,25,26,27] likely reflects developmental differences in cardiac autonomic regulation. Zebrafish studies have shown that the sympatho-vagal balance develops gradually between 2 and 15 days post-fertilization, with autonomic reflexes still incomplete at 5 dpf [35]. At 3 dpf, sympathetic innervation of the heart is just beginning to form, and the cardiac rhythm remains predominantly myogenic, with limited catecholaminergic modulation [36]. As a result, MDPV cannot trigger the systemic sympathetic activation that underlies tachycardia in mammals. Instead, its effects in zebrafish are dominated by direct electrophysiological interference with cardiac ion channels—particularly hERG- and KCNQ1-like repolarizing currents—leading to conduction delay and bradyarrhythmia. Therefore, the zebrafish phenotype most likely reflects direct cardiotoxicity in the absence of mature autonomic compensation, rather than a genuine pharmacological opposition to mammalian outcomes.

Together, these results confirm that MDPV has intrinsic cardiotoxic potential at micromolar concentrations, manifesting as conduction failure rather than simple bradycardia.

### 3.2. Behavioral Effects: Locomotion and Sensorimotor Gating

Increases in locomotor activity following the administration of MDPV and other synthetic cathinones have been reported in multiple experimental paradigms [37,38]. In the present study, however, MDPV induced a significant decrease in basal locomotor activity in 5 dpf larvae, an effect consistent with an overall suppressive rather than stimulatory action of this compound. A similar reduction in locomotor activity was recently reported in 5–6 dpf zebrafish larvae exposed for 3 h to 10–100 µM pyrovalerone, a compound belonging to the same structural family as MDPV [21]. In contrast, exposure of 5 dpf larvae to mephedrone for 3 h resulted in a mild but significant increase in locomotor activity [39]. Variations in exposure duration, cathinone subtype, or zebrafish strain may account for these divergent outcomes. The divergent locomotor effects of MDPV observed between zebrafish and mammalian models likely reflect species- and developmental stage-specific differences in neurophysiology and pharmacokinetics.

The sustained hypoactivity observed after acute MDPV exposure likely reflects a mechanism distinct from the hyperlocomotion typically reported in mammalian models. At low micromolar concentrations, MDPV acts as a potent dopamine transporter (DAT) blocker, producing tonic elevations of extracellular dopamine rather than the phasic surges associated with locomotor stimulation [2]. Such tonic dopaminergic signaling may engage inhibitory feedback circuits or induce behavioral suppression in zebrafish larvae, consistent with species- and stage-specific neurophysiology. In parallel, the marked upregulation of immediate-early genes such as *egr1*, *npas4a*, and the stress-responsive co-chaperone *fkbp5* suggests activation of transcriptional programs involved in synaptic remodeling and glucocorticoid signaling [40,41,42]. These molecular responses may contribute to adaptive dampening of neural excitability under sustained transporter blockade. Together, these findings support a model in which locomotor hypoactivity results from combined dopaminergic dysregulation and stress-related genomic activation, highlighting mechanistic differences between zebrafish and mammalian psychostimulant responses. Some psychoactive compounds, including dextroamphetamine, affect sensorimotor gating in a species-dependent manner, producing enhanced PPI in humans [43] but decreased PPI in rodents [44], consistent with divergent dopaminergic sensitivities between species. The effects of MDPV and related pyrrolidine-containing cathinone derivatives on sensorimotor gating remain poorly characterized and inconsistent [38,45]. Although the dopaminergic agonist apomorphine has been shown to impair PPI in zebrafish larvae via D_2_ receptor activation [46], no previous studies have examined whether psychostimulant compounds can modulate PPI in this model organism. Our results demonstrate for the first time that exposure to dextroamphetamine, a DA releaser agent that induces phasic increases in extracellular DA [47] enhances PPI in 7 dpf larvae, and that this effect is fully reversed by co-exposure with the D_2_ antagonist haloperidol. Thus, the zebrafish response to dextroamphetamine parallels that reported in humans [43].

In contrast, MDPV did not significantly alter PPI across the tested concentration range. Notably, Horsley and colleagues (2018) [48] demonstrated that only at the higher dose tested (4 mg/kg), MDPV decreased PPI in rats, but transiently, when plasma and brain levels were at their peak. Altogether, these findings suggest that MDPV primarily acts through a cocaine-like mechanism as a DAT blocker, producing tonic and sustained elevations in extracellular DA [49,50]. Given its limited direct D_2_ receptor activity [3], MDPV may therefore elicit subtle or secondary D_2_-dependent effects [51] under acute exposure conditions.

### 3.3. Early Gene Expression Responses

Gene expression profiling revealed that short-term MDPV exposure (0.4–400 µM, 2 h) elicited distinct transcriptional signatures across concentrations. The mild but significant up-regulation of *th* (tyrosine hydroxylase) observed between 4 and 400 µM suggests enhanced dopaminergic synthetic activity, likely reflecting a compensatory response to DA reuptake blockade induced by MDPV. Similar transient increases in TH mRNA have been described following acute methamphetamine (METH) administration in discrete dopaminergic nuclei of the rodent brain [52,53]. *th* up-regulation was detected in the brain of adult zebrafish after 3 h of METH exposure [19], and in *Carassius auratus* following treatment with d-amphetamine [54], supporting the view that increased dopaminergic turnover is an early adaptive response to psychostimulant challenge in vertebrates.

Conversely, a transient down-regulation of *slc6a3* (dopamine transporter, DAT) was detected in embryos exposed for 3 h to 40 µM MDPV. This finding is consistent with well-described negative feedback mechanisms reported in mammalian systems, where sustained elevations of extracellular dopamine or prolonged transporter blockade lead to decreased *slc6a3* transcription. Reduced DAT expression has been documented in the brains of postnatal rats after repeated amphetamine administration [55], and in rats chronically exposed to cocaine [56], suggesting that compensatory down-regulation of the transporter constitutes a conserved homeostatic mechanism limiting dopaminergic overstimulation. The concordance between zebrafish and rodent data indicates that even brief MDPV exposure is sufficient to engage transcriptional feedback loops regulating DA synthesis and reuptake.

Acute MDPV exposure triggered rapid and concentration-dependent transcriptional changes typical of psychostimulant action. The marked up-regulation of the immediate-early genes *fosab* and *egr1* at ≥40 µM parallels the activity-dependent genomic response described for dopaminergic and glutamatergic drugs [41,57]. Both genes, induced through Ca^2+^/MAPK signaling, are classical markers of neuronal activation and synaptic remodeling [58].

*npas4a* and *nr4a1* showed biphasic regulation, with the expression down-regulated at low doses and up-regulated at 400 µM, indicating distinct activation thresholds. NPAS4 maintains excitatory–inhibitory balance and limits excitotoxic and inflammatory stress [42,59], whereas NR4A1 integrates dopamine receptor activation with mitochondrial and inflammatory signaling, balancing adaptation and apoptosis [41,42]. Their coordinated induction at high concentrations likely represents recruitment of homeostatic and protective transcriptional pathways in response to intense neuronal activation.

The co-chaperone *fkbp5*, a well-known glucocorticoid-responsive gene, was also significantly up-regulated at 40–400 µM MDPV. FKBP5 is tightly connected to NR4A nuclear receptors and participates in the regulation of inflammatory and neurodegenerative processes [40,42]. The parallel induction of *fkbp5* and *nr4a1* observed here likely reflects coordinated activation of GR- and NR4A-dependent transcriptional networks, linking dopaminergic overactivity with endocrine and immune stress signaling.

Although *kcnh2a* was transiently down-regulated at 40 µM, MDPV did not change the expression of *kcnh6a* or *kcnq1* at any concentration tested. This finding is mechanistically relevant, as *kcnh6a*, rather than *kcnh2a*, encodes the functionally dominant ventricular hERG-like channel mediating IKr in zebrafish [60]. Acute IKr blockade has been reported to produce AV-block phenotypes comparable to those observed here [61], and KCNQ1-dependent IKs contributes to ventricular repolarization and arrhythmia susceptibility [61,62]. Thus, the modest *kcnh2a* down-regulation does not appear to drive the observed cardiotoxic effects, which are more consistent with direct functional interference with repolarizing K^+^ channels than with early genomic remodeling of ion-channel transcripts.

Overall, the combined upregulation of *fosab*, *egr1*, *npas4a*, *nr4a1*, and *fkbp5* defines a conserved transcriptional program integrating neuronal activation, stress adaptation, and inflammatory control. These early molecular events parallel those reported for other psychostimulants in zebrafish and mammals [41,57], underscoring the suitability of the model for elucidating the mechanistic basis of MDPV neurotoxicity.

### 3.4. Integrative Interpretation and Relevance

The combined physiological, behavioral, and molecular data outline a coherent framework of MDPV action in zebrafish embryos. At higher concentrations, MDPV directly compromises cardiac excitability, leading to conduction failure and mortality. However, causality between cardiotoxic endpoints and mortality cannot be inferred in 3 dpf zebrafish embryos, since cutaneous gas exchange allows survival without functional circulation [33]. At sublethal levels, it suppresses locomotion and triggers dopaminergic and stress-related transcriptional responses without disrupting sensorimotor gating. This divergence suggests that molecular activation of dopaminergic pathways precedes overt neurobehavioral deficits, possibly reflecting the predominance of transporter blockade over receptor-mediated signaling during acute exposure.

The preservation of *kcnh6a* and *kcnq1* expression despite severe bradyarrhythmia reinforces the conclusion that MDPV acts as a direct cardiotoxicant targeting repolarization, while neurobehavioral effects emerge at much lower concentrations.

The marked difference between concentrations eliciting neurobehavioral suppression (EC_50_ ≈ 2.5 µM) and those producing atrioventricular conduction block (EC_50_ ≈ 228 µM) likely reflects engagement of distinct mechanistic domains rather than a true toxicological safety margin. At low micromolar levels, MDPV primarily acts as a potent dopamine transporter (DAT) blocker, consistent with its psychostimulant profile [2]. In contrast, substantially higher concentrations may be required to interfere with cardiac repolarization, consistent with studies reporting prolonged field potential duration in human iPSC-derived cardiomyocytes exposed to MDPV [32]. Although our study did not directly assess transporter function or ion-channel currents, these interpretations align with the known pharmacology of pyrrolidine-containing cathinones. Importantly, the zebrafish model isolates these direct electrophysiological effects from systemic factors, particularly hyperthermia and sympathetic overactivation, that markedly lower the cardiotoxicity threshold in mammals.

From a toxicodynamic perspective, comparison of concentration–response relationships across endpoints clarifies MDPV’s functional safety margin. The EC_50_ for suppression of basal locomotor activity (2.51 µM), a proxy for central behavioral or psychostimulant-related effects, was approximately 91-fold lower than the EC_50_ for atrioventricular conduction block (228 µM). This difference indicates that functional cardiac toxicity occurs at concentrations far exceeding those producing neurobehavioral alterations, suggesting that acute exposures sufficient to elicit psychotropic effects remain well below the cardiotoxic threshold in zebrafish. Nonetheless, the steep concentration–response slopes observed for both endpoints imply a relatively narrow dynamic range between behavioral and cardiac domains, consistent with the abrupt cardiovascular collapse reported in severe MDPV intoxications in mammals.

The integration of cardiac, behavioral, and gene-expression endpoints highlights the zebrafish’s ability to disentangle the mechanistic layers of psychostimulant toxicity and to predict mammalian outcomes without confounding them with hyperthermia or systemic stress. Such a multi-level approach upgrades zebrafish from a screening model to a platform capable of identifying conserved signatures of neuro- and cardiotoxicity.

### 3.5. Conclusions and Perspectives

This study demonstrates that zebrafish provide a sensitive and integrative model for assessing the cardiotoxic and neurobehavioral effects of synthetic cathinones. MDPV induced concentration-dependent bradycardia and conduction block, reduced locomotor activity, and rapidly modulated dopaminergic and stress-related genes, without affecting sensorimotor gating. These findings reveal that zebrafish can distinguish between mechanistic components of psychostimulant action and toxicity, offering a valuable system for early hazard identification.

The preservation of *kcnh6a* and *kcnq1* expression despite severe conduction disturbances supports a direct, functional interference of MDPV with repolarizing K^+^ currents rather than transcriptional remodeling, reinforcing its intrinsic cardiotoxic potential. Comparison of concentration–response relationships across endpoints further revealed that behavioral alterations occur at concentrations nearly two orders of magnitude lower than those producing cardiac conduction failure. This quantitative distinction defines a functional safety margin between neurobehavioral engagement and cardiotoxic risk and highlights the translational potential of zebrafish assays for evaluating relative thresholds of adverse drug effects.

Future studies should extend this framework to chronic exposures and in vivo imaging approaches to clarify how acute molecular responses evolve into long-term neurocardiac dysfunction.

## 4. Materials and Methods

### 4.1. Zebrafish Maintenance and Embryo Production

Wild-type short-fin sexually mature adults zebrafish, within the typical standard length range for breeding stock (3.0–3.5 cm), were obtained from a commercial supplier (Exopet, Madrid, Spain) and were maintained under a 12 h: 12 h light/dark cycle and a temperature of 28 ± 1 °C at the Research and Development Center (CID-CSIC) in a recirculating water system (Aquaneering Inc., San Diego, CA, USA).

The day before each experiment, breeding groups composed by three females and two males were transferred to a breeding tank. Fertilized eggs were collected within 30 min post-spawning (lights-on). Only high-quality embryos at the 50%-epiboly stage were selected under a stereomicroscope (Nikon SMZ1500, Champigny-sur-Marne, France). Embryos were transferred to a crystallizing dish containing embryo water [Milli-Q water with 90 mg/L Instant Ocean (Aquarium Systems, Sarrebourg, France) and 0.58 mM CaSO_4_·2H_2_O, pH 6.5–7.0, 750–900 μS/cm conductivity] and incubated at 28 ± 1 °C in a climatic chamber (POL-EKO APARATURA KK350, Wodzisław Śląski, Poland) under a 12 h:12 h light/dark cycle until the start of the experiments at 3 days post-fertilization (3 dpf).

All procedures were approved by the Institutional Animal Care and Use Committees at the CID-CSIC and conducted in accordance with the institutional guidelines under a license from the local government (agreement number 11336).

### 4.2. Chemicals

MDPV (MW = 311.81 g/mol) was synthesized in the University of Barcelona (UB) as hydrochloride salts in a racemic form as previously published [63]. Stock solutions (10 mM) were freshly prepared on the day of each experiment in Milli-Q water. Working solutions were obtained by dilution in embryo water, and the pH was then adjusted to 8.7–9.0 with 1 M NaHCO_3_ to maintain it within the 8.4–9.5 range corresponding to the compound’s pK_a_, thereby ensuring its predominant ionized form throughout the exposure [64]. Dextroamphetamine (or *d*-amphetamine) sulfate was generously provided by Prof. Dr. Camarasa from the University of Barcelona.

### 4.3. Lethality Test

At 3 dpf, embryos were exposed for 24 h to 0, 60, 120, 240, 300, 360, 420, 480, 540, and 600 µM MDPV in 24-well plates (one embryo per well; *n* = 16 per group, four replicates per treatment) with 1.5 mL solution per well. Lethality was evaluated at the end of the exposure period, and mortality was expressed as percentage lethality for each concentration.

Concentration–response data were analyzed using a four-parameter logistic (4PL) model constrained between 0 and 100% lethality (Equation (1)):

$$Y=\frac{100}{1+10^{\left(logEC50-logX\right)\cdot Hill}}$$

where Y is the percentage of lethality, X the MDPV concentration (µM), LC_50_ the concentration producing 50% mortality, and Hill the slope factor.

Model fitting and parameter estimation were performed in Python (SciPy 1.11) using nonlinear least-squares regression. Uncertainty was assessed via nonparametric bootstrap resampling (*n* = 2000 resamples) to derive 95% confidence intervals (CI_95_) for both LC_50_ and Hill slope values.

The fitted model yielded an LC_50_ of 343.8 µM (95% CI: 318.1–369.4 µM) and a Hill slope of 9.81 (95% CI: 6.75–10.00), indicating a steep transition between sublethal and lethal concentrations.

### 4.4. Cardiotoxicity Assessment

#### 4.4.1. Heart Beating Analysis

Embryos at 3 dpf were exposed for 2 h to 60–480 µM MDPV in 48-well plates (one embryo per well, 1 mL solution). To minimize movement during imaging, embryos were briefly anesthetized (0.08 mg/mL tricaine methane-sulfonate [MS-222], 10 s) and embedded in 3% methylcellulose on a depression microscope slide. Cardiac activity was recorded laterally with a high-speed camera (ace U acA1440-220 um, Basler AG, Ahrensburg, Germany) attached to a stereomicroscope (SMZ1500, Nikon, Champigny sur Marne, France) at 100 fps for 20 s using Pylon Viewer 64-Bit software (9.1.0.1614) at 28 ± 1 °C.

Atrial and ventricular beat frequencies were quantified using DanioScope v1.0 (Noldus Information Technology, Wageningen, The Netherlands), which derives heart rate from pixel-intensity fluctuations over time [30]. Atrial and ventricular activity were analyzed separately to assess chamber-specific effects of MDPV. The atrioventricular (AV) ratio, defined as the ratio between atrial and ventricular beat frequencies, was calculated for each embryo as an indicator of conduction efficiency (1.0 = full AV conduction).

To quantify the degree of conduction impairment, the AV ratio was transformed into percentage AV block according to the expression:

$$AV\;block\left(\%\right)=100\times \left(1-\frac{1}{R}\right)$$

where R is the AV ratio. This transformation yields values from 0% (normal conduction) to 100% (complete AV block).

Concentration–response analysis of AV block was performed using a four-parameter logistic (4PL) model constrained between 0 and 100%. The model was fitted to individual replicate data at concentrations ≥ 60 µM using nonlinear least-squares regression in Python (SciPy 1.11). Equation (1) was used, where Y is the percentage of AV block, X the MDPV concentration (µM), EC_50_ the concentration eliciting 50% AV block, and Hill the slope parameter. Model performance and uncertainty were evaluated by nonparametric bootstrap resampling (*n* = 2000 resamples), from which 95% confidence intervals were calculated for both EC_50_ and Hill slope

The fitted model yielded an EC_50_ of 228.3 µM (95% CI: 208.1–253.3 µM) and a Hill slope of 1.45 (95% CI: 1.30–1.62), defining the midpoint of the transition from synchronous atrial–ventricular conduction to high-degree AV block.

#### 4.4.2. Cardiac Mechanical Performance

To comprehensively assess cardiac function, several parameters were quantified from the heart activity recordings, including fractional shortening (FS), stroke volume (SV), cardiac output (CO), and ejection fraction (EF). Together with heart rate, these parameters provide an integrated evaluation of cardiac performance and efficiency in the exposed embryos.

Recorded videos were imported into Fiji (ImageJ, version 1.54p; Java 1.8.0_322, 64-bit), and cardiac parameters were calculated as described by Benslimane et al. [18] (Supplementary Figure S5). Three end-diastolic and three end-systolic frames were selected, and the ventricular long axis, short axis, and area were measured in each frame. The mean of the three measurements was then used for analysis. Since the values obtained were expressed in pixels, they were converted to real units (µM) using a calibration factor, according to the following equations:

Calibration factor (µM/pixel) = Camera pixel size (µM)/Total magnification(1)

Real unit value (µM) = Pixel unit value × Calibration factor(2)

Using these real unit values, the following cardiac parameters were calculated:

Fractional shortening (%) = 100 × (D_d_ − D_s_)/D_d_ (3)

Volume = (1/6) × π × D_L_ × D_S_^2^ (4)

Stroke volume (nL) = End-diastolic volume − End-systolic volume(5)

Cardiac output (nL/min) = Stroke volume (nL/beat) × Heart rate (bpm)(6)

Ejection fraction (%) = (Stroke volume/End-diastolic volume) × 100(7)

where D_d_ is the ventricular diameter in diastole, D_s_ the ventricular diameter in systole, D_L_ the long-axis ventricular diameter, and D_S_ the short-axis ventricular diameter.

### 4.5. Behavioral Assessment

#### 4.5.1. Basal Locomotor Activity

Basal locomotor activity (BLA) was assessed in 5 dpf zebrafish larvae exposed for 2 h to 5 nM–5 µM MDPV. Untreated embryo water served as the control. Recordings were made on the DanioVision platform (Noldus Information Technology, Wageningen, The Netherlands) under near-infrared illumination with temperature maintained at 28 °C. Video tracking was performed using EthoVision XT software (v13 for recording, v16 for analysis).

Locomotor behavior was quantified during three 15 min observation windows (0–15, 45–60, and 105–120 min). For each larva, BLA was defined as the total distance (cm) traveled within each window. Results are presented as percentage of the control values (0 µM).

Concentration–response relationships for the 105–120 min interval were analyzed using a four-parameter logistic (4PL) regression model, fitted to all individual replicates (excluding the 0 µM control, since log(0) is undefined). The model followed the equation:

$$Y=Bottom+\frac{\left(Top-Bottom\right)}{1+10^{\left(logEC50-logX\right)\cdot Hill}}$$

where Y represents the locomotor response (% control), X the MDPV concentration (µM), Top and Bottom the asymptotic plateaus, Hill the slope, and EC_50_ the half-maximal effective concentration.

Nonlinear regression was performed in Python (SciPy 1.11) using bounded least-squares fitting with a maximum of 20,000 iterations. The 95% confidence interval for EC_50_ was obtained by nonparametric bootstrap resampling (1000 stratified resamples per concentration). Derived EC_10_ and EC_90_ values were calculated by inverting the fitted function at 10% and 90% of the response span, respectively. Model reliability was evaluated by inspection of fitted curves and 95% bootstrap prediction bands.

Nonparametric group comparisons were conducted using Kruskal–Wallis tests followed by pairwise post hoc analyses, identifying significant locomotor suppression at 0.5 µM and 5 µM (*p* < 0.05).

#### 4.5.2. Prepulse Inhibition

Prepulse inhibition (PPI) of the acoustic startle response was assessed in 7 dpf larvae, either unexposed (control) or exposed for 30 min to MDPV concentrations ranging from 5 nM to 5 µM, using the Zebra_K+ platform (add-on module for embryos and larvae for Zebra_K [65]). The 7 dpf developmental stage was selected because, at this age, zebrafish possess a fully functional dopaminergic modulatory circuit, allowing reliable measurement of sensorimotor gating [23].

The PPI paradigm consisted of three sequential steps. In the prepulse step, five weak auditory stimuli (1000 Hz, 20 µs, 74.8 dB re 20 µPa) that typically elicited 0–10% startle responses were delivered at 120 s interstimulus intervals (ISI). After a 60 s pause, the pulse step comprised five startle-inducing stimuli (1000 Hz, 4 ms, 93.7 dB re 20 µPa) presented at the same 120 s ISI. Following another 60 s rest period, prepulse–pulse pairs were delivered in the PPI step, with 50 ms and 500 ms prepulse–pulse intervals, and a 120 s ISI between successive pairs.

Dextroamphetamine (5 µM) was included as a positive control, as it represents a prototypical amphetamine-type stimulant with well-documented disruptive effects on PPI mediated by enhanced dopaminergic transmission.

### 4.6. RNA Preparation and qRT-PCR Analysis

Total RNA was extracted from pools of six 3 dpf embryos (control or exposed to 0.4–400 µM MDPV) using TRIzol Reagent (Invitrogen, Carlsbad, CA, USA) [66]. RNA concentration and purity were determined spectrophotometrically (NanoDrop ND-8000, NanoDrop Technologies, Wilmington, DE, USA). After DNase I treatment (Ambion, Austin, TX, USA), RNA was reverse-transcribed with the First Strand cDNA Synthesis Kit (Roche Diagnostics, Mannheim, Germany).

qRT-PCR was performed on a LightCycler 480 System (Roche) using SYBR Green Master Mix (Roche) under the following conditions: 95 °C for 15 min, then 45 cycles of 95 °C for 10 s and 60 °C for 30 s. Each condition included eight biological and three technical replicates. Primers for *th*, *slc6a3*, *fosab*, *egr1*, *npas4a*, *nr4a1*, *fkbp5*, *kcnh2a, kcnh6a*, and *kcnq1* were designed with Primer Express 2.0 (Applied Biosystems) and validated with Primer-BLAST (https://www.ncbi.nlm.nih.gov/tools/primer-blast; accessed on 20 May 2025). Primer sequences and the reference gene *ppiaa* (peptidyl-prolyl isomerase A) are listed in Supplementary Table S2. Relative mRNA levels were normalized to *ppiaa* and fold-changes calculated by the ΔΔCt method [67].

### 4.7. Data Analysis

Statistical analyses were performed using IBM SPSS Statistics v29 (IBM Corp., Chicago, IL, USA), and visualizations with GraphPad Prism 10 (GraphPad Software, San Diego, CA, USA). Data normality was assessed by the Shapiro–Wilk test. Results are presented as mean ± SEM (parametric data) or median [IQR] (non-parametric data).

Nonparametric bootstrap resampling (stratified by concentration, 2000 iterations) was used to derive 95% percentile confidence intervals for nonlinear curve parameters (EC_50_/LC_50_ and Hill slope), providing robust estimation of uncertainty without relying on distributional assumptions for residuals or replicate values. Nonlinear regression was performed using a four-parameter logistic model fit by bounded least-squares minimization.

Cardiotoxicity data were analyzed by mixed repeated-measures ANOVA followed by Tukey’s post hoc test to evaluate concentration and chamber effects (atrium vs. ventricle). When appropriate, separate one-way ANOVAs were run for each chamber.

Locomotor activity data (total distance traveled) were analyzed using the Kruskal–Wallis test since normality assumptions were not met. Statistical significance was set at *p* < 0.05.

## Data Availability

The original contributions presented in this study are included in the article/Supplementary Material. Further inquiries can be directed to the corresponding author.

## Supplementary Materials

The following supporting information can be downloaded at: https://www.mdpi.com/article/10.3390/ijms27010059/s1.
