# Supplementary material for: Integrated Assessment of the Cardiotoxic and Neurobehavioral Effects of 3,4-Methylenedioxypyrovalerone (MDPV) in Zebrafish Embryos
Source: Int J Mol Sci. 2025 Dec 20;27(1):59. doi: 10.3390/ijms27010059 (PMC12785441; doi:10.3390/ijms27010059)
Supplement: Supplementary file 1 [file ijms-27-00059-s001.zip › Supplementary Material.pdf]

Supplementary Material

Integrated Assessment of the Cardiotoxic and Neurobehavioral Effects of 3,4-Methylenedioxypyrovalerone (MDPV) in Zebrafish Embryos

Ouwais Aljabasini, Niki Tagkilodou, Juliette Bedrosiantz, Eva Prats, Raul Lopez Arnau, Demetrio Raldua

Supplementary Tables

Supplementary Table S1. Statistical analysis of gene expression after 2 h MDPV exposure in 3 dpf zebrafish embryos. ΔΔCt values were normalized within each experiment.

<u>Table S1A:</u>

Gene	Statistical test	Statistic	Overall *p*-value	[MDPV]	Post-hoc test	*p*-value
th	ANOVA	$F(3,26) = 4.869$	0.008	0.4 µM	Dunnett	0.196
				4.0 µM	Dunnett	0.08
				40 µM	Dunnett	0.09
slc6a3	ANOVA	$F(3,26) = 3.966$	0.019	0.4 µM	Dunnett	0.092
				4.0 µM	Dunnett	0.106
				40 µM	Dunnett	0.007
fosab	ANOVA	$F(3,26) = 44.381$	2.28×10^{-10}	0.4 µM	Dunnett	0.262
				4.0 µM	Dunnett	0.092
				40 µM	Dunnett	7.18×10^{-8}
egr1	ANOVA	$F(3,25) = 55.567$	3.37×10^{-11}	0.4 µM	Dunnett	0.947
				4.0 µM	Dunnett	0.698
				40 µM	Dunnett	1.88×10^{-8}
npas4a	ANOVA	$F(3,25) = 5.829$	0.004	0.4 µM	Dunnett	0.089
				4.0 µM	Dunnett	0.001
				40 µM	Dunnett	0.0029
nr4a1	ANOVA	$F(3,25) = 12.974$	2.63×10^{-5}	0.4 µM	Dunnett	3.17×10^{-4}
				4.0 µM	Dunnett	2.24×10^{-5}
				40 µM	Dunnett	0.159
fkbp5	ANOVA	$F(3,26) = 11.167$	6.83×10^{-5}	0.4 µM	Dunnett	0.095
				4.0 µM	Dunnett	0.782
				40 µM	Dunnett	0.006
kcnh2a	KW	$H(3) = 14.838$	0.002	0.4 µM	Dunn	0.977
				4.0 µM	Dunn	0.426
				40 µM	Dunn	1.45×10^{-4}
kcnh6a	KW	$H(3) = 2.416$	0.491	0.4 µM	Dunn	0.413
				4.0 µM	Dunn	0.239
				40 µM	Dunn	0.999
kcnq1	KW	$H(3) = 4.914$	0.178	0.4 µM	Dunn	0.762
				4.0 µM	Dunn	0.258
				40 µM	Dunn	0.116

Table S1B:

Gene	Statistical test	*t*(df)	*p*-value (two-tailed)
th	Unpaired *t*-test	−5.011 (14)	1.91×10^{-4}
slc6a3	Welch-corrected *t*-test	0.986 (8.334)	0.352
fosab	Unpaired *t*-test	−12.908 (14)	3.65×10^{-9}
egr1	Unpaired *t* -test	−7.033 (14)	5.93×10^{-6}
npas4a	Unpaired *t* -test	−6.801 (14)	8.59×10^{-6}
nr4a1	Unpaired *t* -test	−9.458 (14)	1.85×10^{-7}
fkbp5	Welch-corrected *t* -test	−9.915 (7.955)	1.04×10^{-7}
kcnh2a	Unpaired *t* -test	0.368 (14)	0.719
kcnh6a	Unpaired *t* - test	−0.826 (14)	0.423
kcnq1	Unpaired *t* -test	−1.077 (14)	0.3

Normality of residuals was evaluated using the Shapiro–Wilk test. In Experiment 1, normally distributed datasets were analyzed using one-way ANOVA followed by Dunnett's test, whereas Kruskal–Wallis tests followed by Dunn's post-hoc were used when normality was not met. In Experiment 2, comparisons were limited to 400 µM vs. control using unpaired t-tests (with Welch correction where variances were unequal). All ΔΔCt values were normalized within each experiment to avoid batch effects.

Supplementary Table S2. List of primers used for the qPCR

Gene	ZFIN Acc number	GenBank Acc number		Sequence	Amplicon length
egr	ZDB-GENE-980526-320	NM_131248	FW	5'-TAAGATCCACATGCGGCAGA	118 bp
			RV	5'-AGAGGGGTAGCTGGACACTG	
fkbp5	ZDB-GENE-030616-630	NM_213149.2	FW	5'-TCAACCTGCAAACCCTGGTA	135 bp
			RV	5'-AACGAACAAGCGGGTCTGAA	
fosab	ZDB-GENE-031222-4	NM_205569	FW	5'-ATGGCATTGCAGGGCTATGG	80 bp
			RV	5'-GGCAGGCATGTATGGTTCAGA	
kcnh2a	ZDB-GENE-070912-699	NM_001042722.2	FW	5'-ACCCACACTTGTGATGTTGTC	81 bp
			RV	5'-TGGCATACATACTTGAAGCCT	
kcnh6a	ZDB-GENE-040702-5	NM_212837.1	FW	5'-TGTGGTTAGCTGTTTGACCCA	76 bp
			RV	5'-ACACACAGATTCATTTGGTGCC	
kcnhq1.1	ZDB-GENE-061214-5	NM_001042722.2	FW	5'-CAGCTGAGTCACCAACGGAG	95 bp
			RV	5'-TGATGCTGAACCTGAGCCAAA	
npas4a	ZDB-GENE-060616-396	NM_001045321	FW	5'-AGCAGGAAAAATAGGGACAGGG	96 bp
			RV	5'-ACGTTAGCATGTCGACACCA	
nr4a1	ZDB-GENE-040704-11	NM_001002173.1	FW	5'-GGTGTGAGATCCAGTGCAGC	94 bp
			RV	5'-GGTACCGCACACTAAGCCAG	
ppiaa	ZDB-GENE-030131-8556	NM_212758.2	FW	5'-TTGTGGAGGGCTTGGATGTC	169 bp
			RV	5'-GGCAGGATTTCGGAAAGGGA	
slc6a3	ZDB-GENE-010316-1	NM_131755.1	FW	5'-GCCTGGTTTTACGGAGTGGA	110 bp
			RV	5'-AGGAAACACGGGCTCACAAA	
th	ZDB-GENE-990621-5	NM_131149.1	FW	5'-GCTTTGTGGACGCTACTGAG	72 bp
			RV	5'-CGAACCGCACAGAAAACGG	

Supplementary Figures

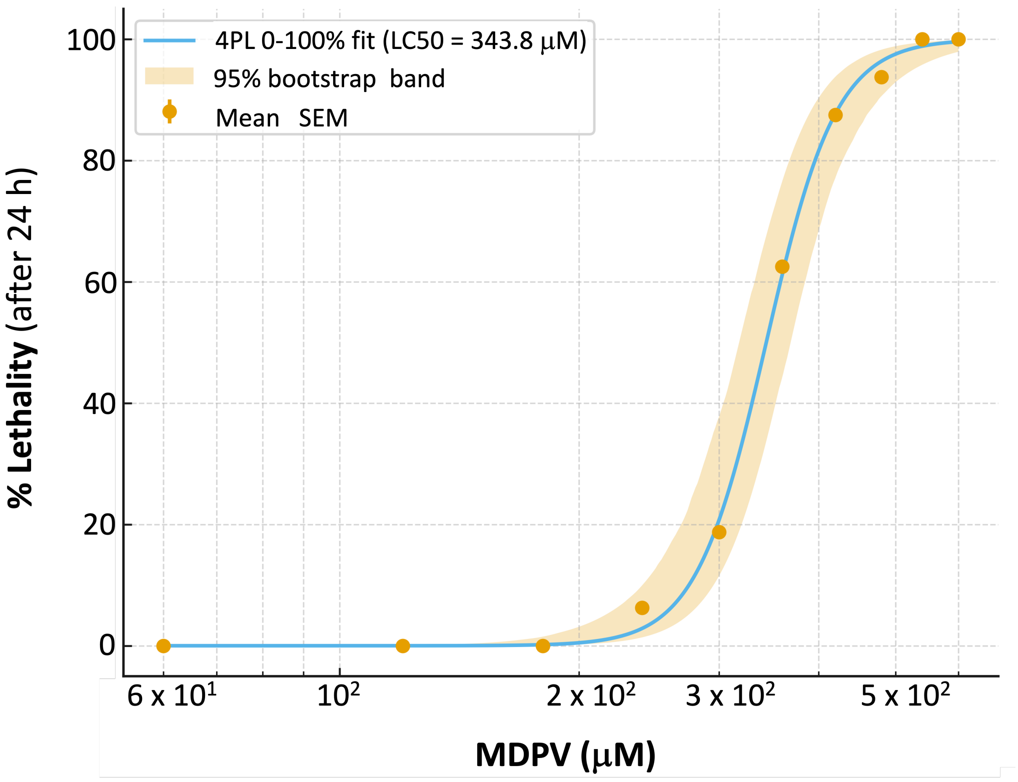

Supplementary Figure S1. MDPV lethality in 3 dpf zebrafish embryos after 24 h exposure. Percent lethality (means ± SEM across four independent experiments) was fitted with a four-parameter logistic model constrained between 0–100%. The fit yielded LC_{50} = 343.8 (95% bootstrap CI: 318.1–369.4) with a Hill slope of 9.81. Shaded band: 95% bootstrap prediction band.

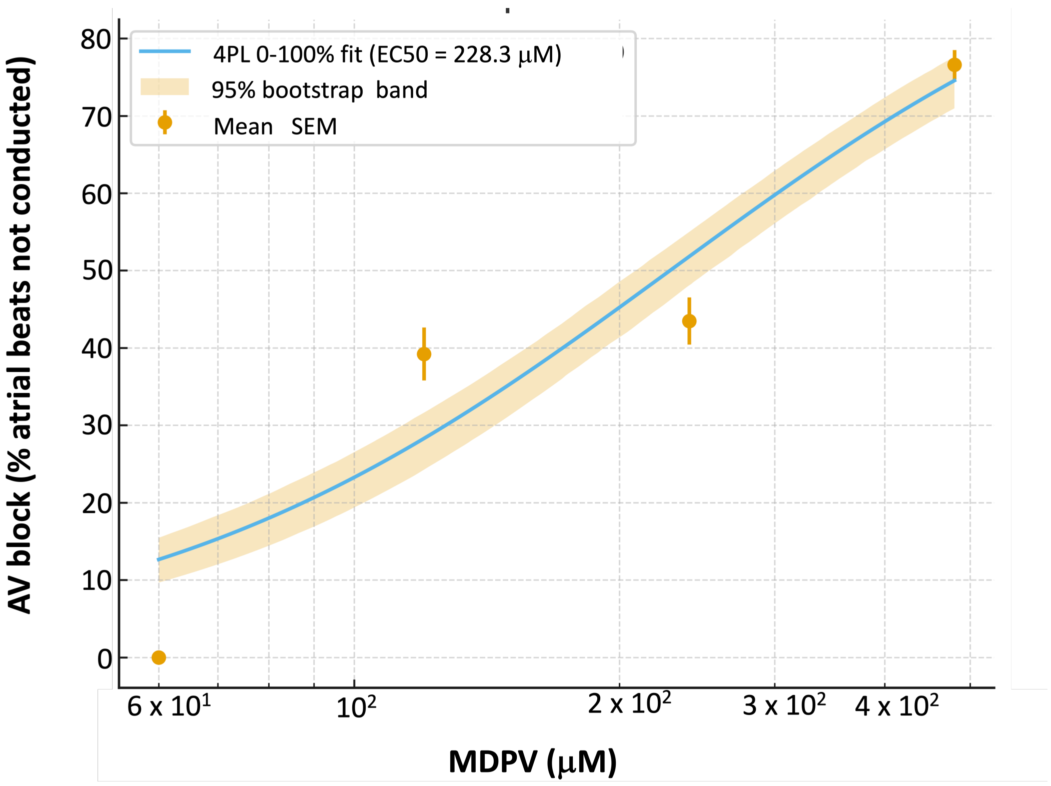

Supplementary Figure S2. AV block induced by MDPV in 3 dpf zebrafish embryos after 2 h exposure. Conduction failure was quantified from the atrial:ventricular (AV) beat ratio and expressed as % AV block = 100 × (1 − 1/R), where R is the AV ratio (1 = no block). Data (means ± SEM) are shown for 60–480 µM. A four-parameter logistic model constrained to 0–100% yielded EC_{50} = 228.3 µM (95% bootstrap CI: 208.1–253.3 µM) and Hill slope = 1.45. Shaded band: 95% bootstrap prediction band. Replicates: 60 µM (n=27), 120 µM (n=40), 240 µM (n=31), 480 µM (n=34).

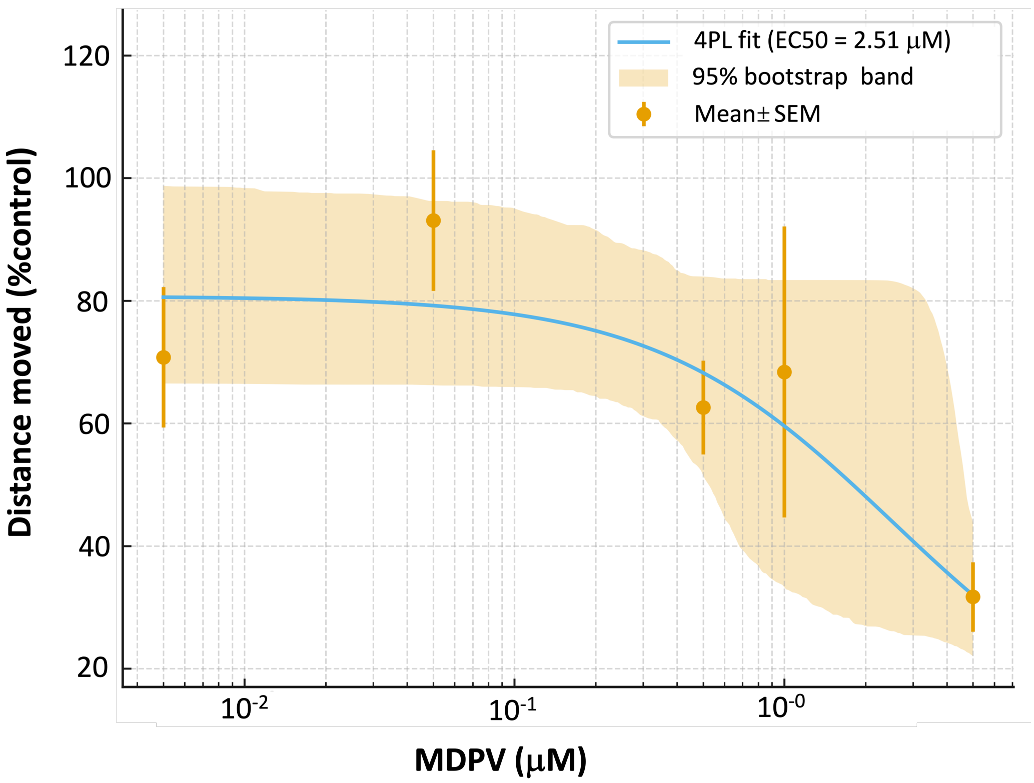

Supplementary Figure S3. Concentration-dependent effects of MDPV on basal locomotor activity (BLA) in 5 dpf zebrafish larvae. Larvae were exposed for 2 h to 5 nM–5 μM MDPV. Locomotor activity was quantified as total distance moved during 105–120 min and expressed as % of the control group (mean ± SEM; n per group: 0 μM = 52, 0.005 μM = 32, 0.05 μM = 32, 0.5 μM = 52, 1 μM = 20, 5 μM = 52). The concentration–response relationship was fitted to a four-parameter logistic model using replicate-level data (excluding 0 μM), yielding EC_{50} = 2.51 μM with 95 % bootstrap CI = 0.38–5.08 μM and a Hill slope = −0.98. The fitted top and bottom plateaus were ~80.7 % and 7.3 % of control, respectively. The shaded band represents the 95 % bootstrap prediction band of the fitted curve.

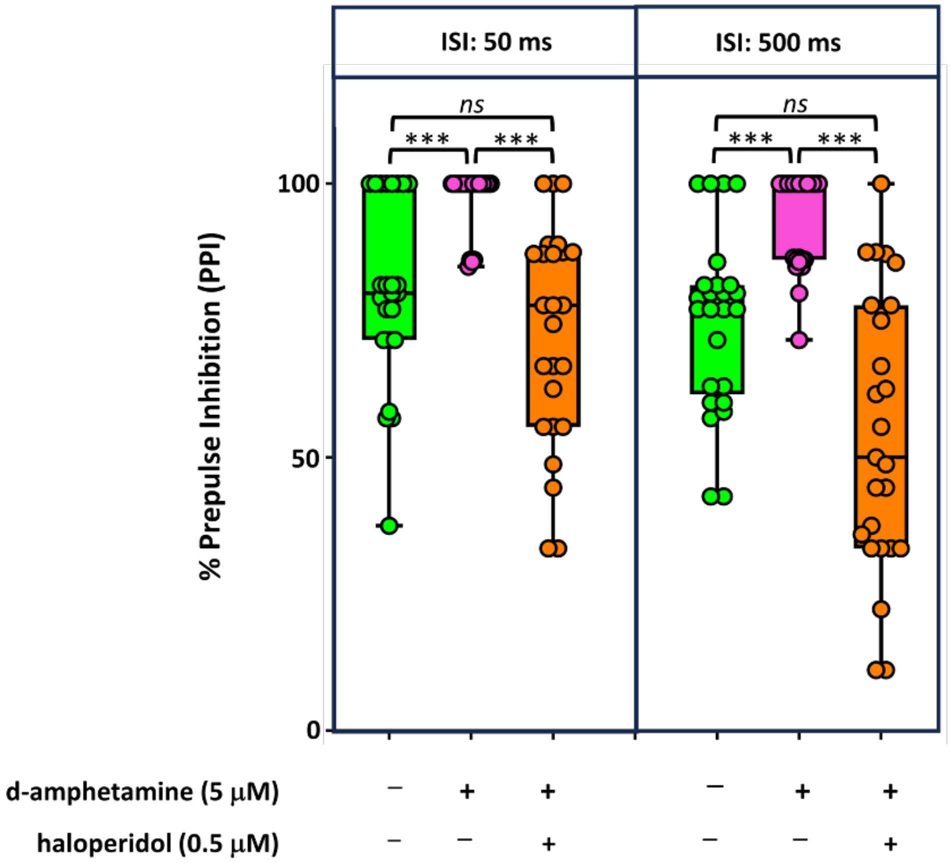

Supplementary Figure S4. Effects on prepulse inhibition (PPI) of the acoustic startle response (ASR) in zebrafish 7 days post-fertilization zebrafish larvae exposed to d-amphetamine. Exposure to 5 μM d-amphetamine for 30 min reduces PPI, and a 20-minute pre-treatment with 0.5 μM haloperidol followed by co-exposure with 5 μM d-amphetamine rescues the effect of d-amphetamine on PPI ($N_{control}$ = 35 larvae; $N_{amphetamine}$ = 39 larvae; $N_{amphetamine+haloperidol}$ = 45 larvae). Significance levels: **$p < 0.01$, ***$p < 0.001$. Prepulse-pulse intervals: 50-ms and 500-ms

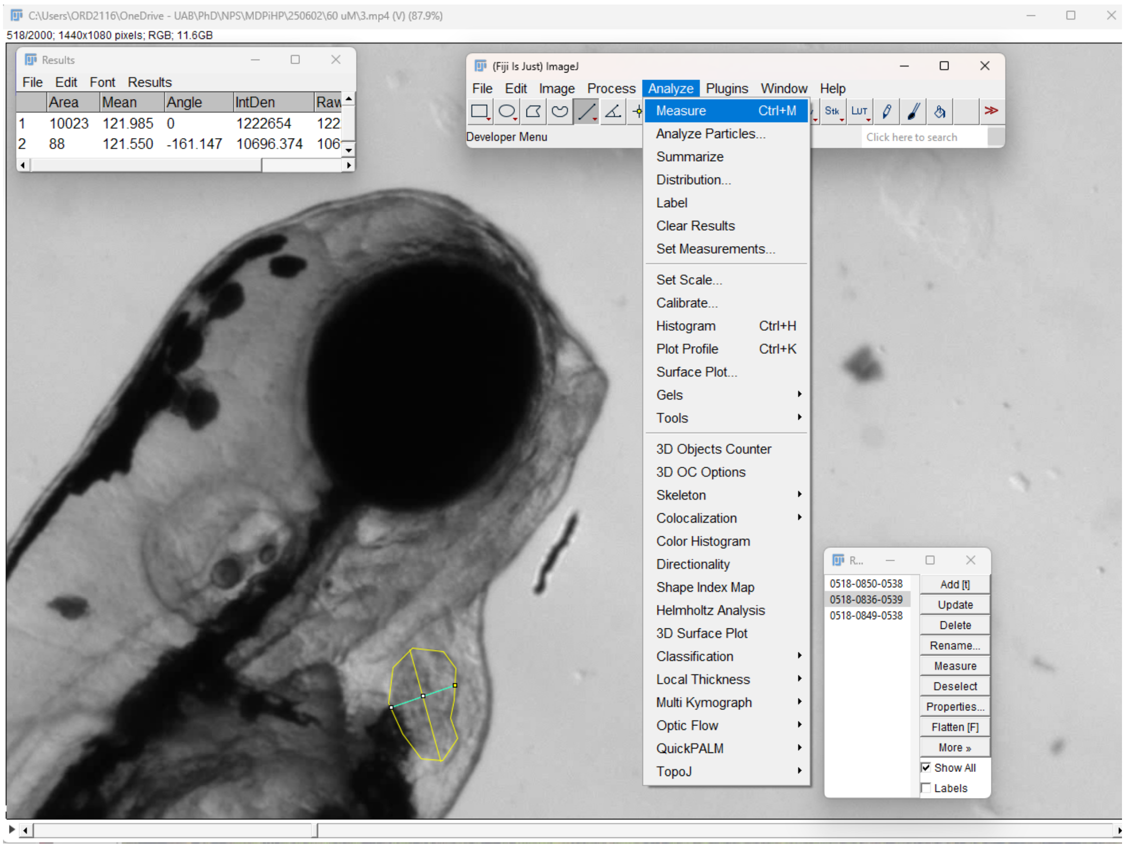

Supplementary Figure S5 ImageJ user interface showing steps used for cardiac parameter measurement in 3 dpf zebrafish embryo.
